# Lack of a skeletal muscle phenotype in adult human bone marrow stromal cells following xenogeneic-free expansion

**DOI:** 10.1186/s13287-020-1587-0

**Published:** 2020-02-22

**Authors:** Dominik Barisic, Marita Erb, Marie Follo, Dahlia Al-Mudaris, Bernd Rolauffs, Melanie L. Hart

**Affiliations:** 1 G.E.R.N. Center for Tissue Replacement, Regeneration and Neogenesis, Department of Orthopaedics and Trauma Surgery, Medical Center - University of Freiburg, Faculty of Medicine, University of Freiburg, Freiburg, Germany; 2 Department of Medicine I, Medical Center - University of Freiburg, Faculty of Medicine, University of Freiburg, Freiburg, Germany

**Keywords:** Mesenchymal stromal cells, MSCs, Myogenesis, Neurogenesis, Coronin 1b, GAP-43, Plasma, Platelet lysate, Lineage determination, Xenogeneic-free

## Abstract

**Background:**

Many studies have elegantly shown that murine and rat bone marrow-derived mesenchymal stromal cells (bmMSCs) contribute to muscle regeneration and improve muscle function. Yet, the ability of transplanted human bmMSCs to manifest myogenic potential shows conflicting results. While human adipose- and umbilical cord-derived MSCs can be differentiated into a skeletal muscle phenotype using horse serum (HS), bmMSCs have only been shown to differentiate towards the skeletal muscle lineage using a complex mixture of cytokines followed by transfection with notch intracellular domain.

**Methods:**

Since xenogeneic-free growth supplements are increasingly being used in the expansion of bmMSCs in clinical trials, we investigated the effects of human plasma and platelet lysate (P/PL) on the expression of neuromuscular markers and whether P/PL-expanded human bmMSCs could be differentiated towards a skeletal myogenic phenotype. Neuromuscular markers were measured using the highly sensitive droplet digital polymerase chain reaction for measuring the expression of Myf5, MyoD, MyoG, ACTA1, Desmin, GAP-43, and Coronin 1b transcripts, by performing immunofluorescence for the expression of Desmin, GAP-43, and MEF2, and flow cytometry for the expression of CD56/neural cell adhesion molecule (NCAM).

**Results:**

Despite that bmMSCs expressed the myogenic regulatory factor (MRF) MEF2 after expansion in P/PL, bmMSCs cultured under such conditions did not express other essential MRFs including Myf5, MyoD, MyoG, or ACTA1 needed for myogenesis. Moreover, HS did not induce myogenesis of bmMSCs and hence did not induce the expression of any of these myogenic markers. P/PL, however, did lead to a significant increase in neurogenic GAP-43, as well as Desmin expression, and resulted in a high baseline expression of the neurogenic gene Coronin 1b which was sustained under further P/PL or HS culture conditions. Fetal bovine serum resulted in equally high levels of GAP-43 and Coronin 1b. Moreover, the proportion of CD56/NCAM-positive bmMSCs cultured in P/PL was 5.9 ± 2.1.

**Conclusions:**

These data suggest that P/PL may prime a small portion of bmMSCs towards an early neural precursor cell type. Collectively, this shows that P/PL partially primes the cells towards a neurogenic phenotype, but does not prime adult human bmMSCs towards the skeletal muscle lineage.

## Background

Skeletal muscle constitutes approximately 40% of the average human body mass and therefore represents one of the most abundant tissues in the human body [1]. Healthy skeletal muscle has a robust capacity for regeneration following injury due to its intrinsic ability of resident stem cells or satellite cells to proliferate, differentiate, and fuse to form new or repair existing myofibers upon injury. This regenerative response is hindered when substantial skeletal muscle loss occurs upon traumatic injuries or in degenerative pathologies, myopathies, and aging [2]. Developing effective treatments for the regeneration of musculoskeletal disorders is a major focus of cell-based regenerative medicine.

Cell therapy efforts to regenerate skeletal muscle are evident by the increasing number of ongoing and upcoming clinical trials. Many cell types have been proposed to regenerate skeletal muscle including cells of myogenic or non-myogenic origins [2–5]. Significant pre-clinical and clinical efforts have focused on the use of bone marrow-derived mesenchymal stem cells (bmMSCs). However, studies using human bmMSCs to regenerate skeletal and cardiac muscle have resulted in varying degrees of success [2, 6, 7]. Thus, clinical trials, the majority of which used autologous bone marrow mononuclear cells (BMMNC) as a cellular means of therapy, have found only a modest benefit. Autologous BMMNCs were used to treat muscular dystrophy patients and demonstrated safety, efficacy, and some clinical improvement, but only a few patients demonstrated muscle regeneration [8, 9]. The most widely used treatment of bmMSCs has been in cardiac repair [7, 10–13]. Cardiomyopathy clinical meta-analysis studies have shown that both bmMSC and BMMNC treatments are feasible and safe. However, the overall efficacy and clinical endpoints were largely inconsistent. Such inconsistencies could be attributed to the dosage of injected cells, number of injections, delivery route, cell survival in situ after injection, endpoints examined, or timing of follow-up, but could also simply be the result of how the MSCs were prepared or expanded prior to application, as we have shown that expansion conditions can impact MSC lineage determination and prime the cells towards certain phenotypes [14, 15].

Hence, many studies have shown that murine and rat bmMSCs contribute to muscle regeneration and can even improve muscle function [16–30], and some have suggested via secretome effects, but the ability of how human bmMSCs manifest myogenic potential is still controversial. Resolving this controversy is critical for understanding the clinical potential and limitations of bmMSC transplantation. Therefore, understanding if human bmMSCs contribute to skeletal muscle myogenesis through, for example, myogenic differentiation will aid in the development of bmMSC-based regenerative cellular therapies.

The ability of MSCs to differentiate in vitro towards the myogenic lineage has been reported by several research groups. Different sources of MSCs were used including placenta [31], umbilical cord [32], and adipose tissue [33, 34]. However, fetal bovine serum (FBS) was consistently used for the expansion. Moreover, while human adipose- (ADSC) and umbilical cord-derived (ucMSCs) MSCs were shown to be capable of differentiating into a skeletal myogenic phenotype using horse serum (HS) following expansion in FBS-containing media [32–34], human bmMSCs have only been shown to differentiate into skeletal muscle lineage cells in vitro using a mixture of cytokines including basic fibroblast growth factor (bFGF), platelet-derived growth factor (PDGF), forskolin, and neuregulin, followed by transfection with the notch intracellular domain and subsequent treatment with HS or MSC supernatant [18] or by using CD45^−^GlyA^−^ bmMSCs cultured in the presence of vascular endothelial growth factor (VEGF), bFGF, and insulin-like growth factors (IGF-1) in FBS-containing media [19]. Although this shows that human bmMSCs are capable of being differentiated towards a skeletal muscle phenotype, this approach may not be clinically feasible and effective alternatives would be greatly beneficial.

Since MSCs are present in extremely low concentrations in the tissues of origin (frequency in the bone marrow is 0.001–0.01%) [35], expansion appears to be necessary to obtain therapeutic cell doses. Due to the safety, ethical, and regulatory concerns involved in the use of FBS, more and more pre-clinical and clinical trials are converting to xenogeneic-free cultures [36, 37]. Human serum (S), plasma (P), or platelet by-products such as platelet lysate (PL) are increasingly being used as alternative replacement growth supplements. Remarkably, human PL is the preferred human supplement for expanding bmMSCs in clinical trials [38] since it was first proposed for use in 2005 as it contains growth factors such as bFGF and PDGF essential for proliferation of MSCs with low batch-to-batch variability [39–42]. Moreover, it has been shown to contain high levels of other growth factors essential for differentiation of bmMSCs into myoblasts [18, 19] including bFGF, PDGF, VEGF, and IGF-1 [39–42]. Further characterization of xenogeneic-based products on MSCs are therefore critical to better understand the resulting cellular phenotypes and for advancing MSC-based therapies.

We previously showed that P/PL is capable of inducing a smooth muscle-like phenotype in human bmMSCs [14, 15]. The goal of the present study was to measure whether human P/PL alone was also sufficient in inducing a skeletal myogenic and neurogenic phenotype. We compared these results to the expansion of human bmMSCs in human P/PL followed by differentiation in standard horse serum-containing media commonly used to differentiate ADSCs, ucMSCs, and C2C12 cells into skeletal muscle cells [18, 32–34, 43]. The question was whether expansion alone or the combination of expansion followed by commonly used induction protocols would lead to differentiation of bmMSCs or prime the cells towards a skeletal myogenic precursor cell type. To the best of our knowledge, the effect of combined human P and PL (P/PL) on the expression of skeletal muscle markers in human bmMSCs has never been investigated. As (micro) droplet digital polymerase chain reaction (ddPCR) has been shown to be advantageous compared to real-time quantitative PCR due to its increased accuracy, reproducibility, and sensitivity [44–46] and can accurately quantify up to one copy of a gene [45], we investigated the expression of (neuro)muscular markers using ddPCR (Myf5, MyoD, MyoG, Acta 1, Desmin, GAP43, and Coronin 1b), immunofluorescence (MyoD, MyoG, Desmin, GAP-43, MEF2), and flow cytometry (neural cell adhesion molecule NCAM/CD56) to help elucidate whether human bmMSCs express (neuro)myogenic genes and proteins as expression of these genes has been shown to be necessary for differentiating myogenic cells into a functional phenotypic cell. The rationale to include (neuro)muscular markers was that MSCs have been proposed for treatment of various types of neuropathies and muscle atrophies that can lead to degeneration of motor neurons and loss of muscle fibers [27, 47–49].

## Methods

### Isolation, expansion, and cultivation of human MSCs

Human bone marrow was collected from up to 9 different male and female donors ranging in age from 24 to 87 years old obtained from the proximal femur during routine hip replacement at the University Medical Center Freiburg, Clinic for Orthopedics and Trauma Surgery according to institutional approval from the University of Freiburg ethics committee (#75/17) and with written informed donor consent. Similar to our previous studies [14, 15, 50–52], the bone marrow was washed with PBS, centrifuged at 150×*g* (10 min at room temperature); the supernatant was discarded; and the cells were resuspended in PBS. MSCs were isolated using a Ficoll® density gradient fractionation (density 1.077 g/mL, GE Healthcare Life Sciences; 150 g, 30 min, room temperature). The mononuclear cell layer was harvested, washed with PBS, and seeded in T75 flasks (BD Falcon). For the expansion of the MSCS with xenogeneic-free medium, Dulbecco’s Modified Eagle Medium (DMEM) containing low glucose (Sigma-Aldrich, Taufkirchen, Germany), 1000 IU heparin (Carl Roth, Karlsruhe, Germany), 25 mmol/L HEPES (Lonza, Basel, Switzerland), 1% penicillin-streptomycin (Life Technologies, Darmstadt, Germany). 2 mmol/L L-glutamine (Lonza), 5% human plasma (P, TCS Biosciences, Buckingham, UK), 5% human pooled platelet lysate (PL, 1x10(8) platelets/ml medium, Blood Donation Center, Freiburg, Germany) was used. For the expansion with xenogeneic-containing medium, 10% FBS (Biochrom AG, Berlin, Germany) was used instead of P/PL. After 24 h of incubation, the media were discarded and replaced to remove unattached cells and media were changed twice a week. After 5–7 days, when MSCs were 70–80% confluent, the cells were removed with Accutase (Sigma-Aldrich), counted and re-seeded in xenogeneic-containing or xenogeneic-free medium (passage 1, density 1.5 × 10^5^ cells per flask). At passage 2 (day 0), when the cells reached a density of approximately 70%, MSCs were seeded onto 12-well plates (Greiner BioOne) at a density of 3000 cells/cm^2^ and further cultured for 0, 3, 7, or 14 days in xenogeneic-containing or xenogeneic-free medium described above. In differentiation experiments, DMEM was supplemented with 5% horse serum, 0.1 μM dexamethasone (Sigma-Aldrich), and 50 μM hydrocortisone (Sigma-Aldrich) similar to [32–34]. Media were changed twice a week.

### Positive controls

Human SJCRH30 cells (ATCC), also known as *RC13 cells*, derived from the bone marrow of a pediatric patient with rhabdomyosarcoma were used as a positive control as these cells show ultrastructural elements of primitive skeletal muscle differentiation, express MyoD and MyoG, and have been used a model of human myoblasts [53, 54]. The average diameter of these cells is 10 μm [55], as opposed to MSCs which are 20–30 μm in diameter [56]. *SJCRH30 cells* were expanded in RPMI 1640 (Sigma-Aldrich), 10% FBS (Biochrom AG), and 1% penicillin-streptomycin (Life Technologies).

Adult skeletal muscle was obtained during total knee replacement surgery at the University Medical Center Freiburg, Clinic for Orthopedics and Trauma Surgery according to institutional approval from the University of Freiburg ethics committee (#75/17) and written informed donor consent. CaCo2 cells were expanded in DMEM high glucose (Sigma-Aldrich), 10% FBS (Biochrom), and 1% penicillin-streptomycin (Life Technologies).

### Flow cytometry analysis of CD56 expression

The expression of the cell surface antigen CD56 on bmMSCs was analyzed by flow cytometry at passage 4. Cells were detached by mild proteolysis (Accutase, Sigma-Aldrich), centrifuged at 500*g* for 5 min at 4 °C, and 5 × 10^5^ cells were resuspended in 50 μL BD Brilliant Stain Buffer (BD Biosciences, USA). Cells were then stained with PE Dazzle 594 anti-human CD56 (NCAM) antibody (Biolegend, USA) and the viability dye Ghost Dye Red 780 (Tonbo Biosciences, CA, USA) for 30 min at 4 °C in the dark. After the incubation time, samples were washed three times with PFEA buffer and centrifuged at 350*g* for 7 min at 4 °C. The cells were then resuspended in 200 μL of ice cold FACS buffer and kept in the dark on ice until analysis. All data was visualized using the BD LSR Fortessa (BD Biosciences) and analyzed using BD FACSDiva 8.0.1 (BD Biosciences) and FlowJo (Treestar Inc., Ashland, OR).

### Droplet digital PCR

Total RNA was extracted according to the manufacturer’s instructions using the RNeasy Plus Micro Kit (Qiagen) with an elution volume of 14 μL with the QIAcube. Following the isolation, RNA concentration was determined by measuring optical density at 260 nm with the NanoDrop spectrophotometer (Thermo Fisher). RNA was reversely transcribed into cDNA according to the manufacturer’s protocol with oligo(dT) and random hexamer primers using the Advantage RT-for-PCR Kit (Clontech). Sixty nanograms of RNA was used for each sample on each day of measurement for reverse transcription.

Droplet digital PCR (ddPCR) was performed using a C1000 Touch™ thermal cycler with gradient function (BioRad). The expression levels of myogenic differentiation markers (MyoD, MyoG, Myf5, Desmin, ACTA1) and neurogenic markers (Coronin 1b, GAP-43) were analyzed by performing absolute quantification experiments using the QX200 Droplet Digital PCR (ddPCR) system (Bio-Rad). All oligonucleotide primers and hydrolysis probes were purchased from BioRad for ddPCR assays: MyoD, MyoG, Myf5, Desmin, ACTA1, Coronin 1b, GAP-43, and GAPDH. Probes were labeled with either a FAM or a HEX dye.

The total reaction volume of 22 μL was comprised of 1.1 μL of each PrimePCR ddPCR Expression Probe Assay, 11 μL ddPCR supermix (ddPCR supermix for probes no dUTP, BioRad), 5 ng cDNA, and 2.2 μL DNAase/RNAse-free water (Qiagen). Assays were used in duplex or singleplex reactions. After pipetting the reaction mix into a 96-well plate, droplets were generated using the QX200 automated droplet generator (BioRad). The droplet emulsion was transferred to another 96-well plate and heat-sealed with pierceable foil heat seal (BioRad) at 180 °C for 5 s using the PX1 plate sealer (BioRad). After placing the 96-well plate into the thermal cycler, PCR was performed using the following temperature profile: (1) polymerase activation at 95 °C for 10 min, (2) 40 cycles of denaturation at 94 °C for 30 s, (3) annealing at 55 °C for 1 min, and (4) denaturation at 98 °C for 10 min. After the thermal cycling, PCR products were kept at 4 °C until digital droplet readout. The fluorescence of the droplets was measured by the QX200 Droplet Reader (Bio-Rad) and subsequently analyzed using QuantaSoft Software (version 1.6.6; Bio-Rad). The software quantified the number of HEX and FAM positive and negative droplets and automatically calculated the target concentration for each HEX and FAM labeled target gene in copies per microliter. Data normalization was achieved by the standardized RNA amount for reverse transcription, the standardized cDNA amount in the reaction volume, and the amount of the expression levels of the reference gene GAPDH.

### Immunofluorescence

MSCs at passage 3 were plated onto 8-well chamber slides (Sarstedt, Nümbrecht, Germany) at a density of 3000 cells/cm^2^ (total of 2400 cells/well) and cultured in differentiation media. On days 0, 14, and 28, immunofluorescent staining was carried out. For this process, cells were fixed with 4% paraformaldehyde (PFA; Electron Microscopy Sciences), washed twice with DPBS, and permeabilized with 0.1% Triton X 100 (Carl Roth, Karlsruhe) for 15 min at room temperature. Cells were then washed twice with DPBS, blocked for 45 min with 1% bovine serum albumin (BSA; Sigma-Aldrich), and incubated with the following primary rabbit antibodies: 1:100 anti-Desmin (Novus Biologicals), 1:400 anti-GAP-43 (Novus Biologicals), and 1:250 anti-MEF2 (Thermo Fisher) at 4 °C overnight. After washing with DPBS, cells were then incubated with 1:1000 Alexa Fluor 568-conjugated goat anti-rabbit (Thermo Fisher) for 60 min at room temperature. Following two washing steps with DPBS, fluoroshield mounting medium with DAPI (Abcam) was applied and microscopy images taken thereafter. As a negative control, cells were incubated with secondary antibody alone. Digital microscopic images recorded in black and white and colorized to green with an exposure time of 1–1.5 s were taken with a CCD camera attached to an Axiovert M200 (Carl Zeiss, Jena, Germany) microscope at magnifications indicated. The percentage of positive cells was determined by manual counting the ratio of positive immunofluorescent cells to DAPI positive nuclei. Semi-quantitative analysis of the relative protein expression intensity was examined and scored by three individuals independently on a scale of low to high intensity defined as follows: grade 0 for less than 10%, grade 1 for 10–50%, grade 2 for 50–80%, and grade 3 for more than 80% of reactive cells.

### Statistical analysis

Data is presented as the average ± the standard deviation of the mean (SEM). Statistical differences were calculated with SigmaPlot 11.0 (Systat Software) using ANOVA on ranks and a Dunn’s post hoc test when comparing multiple groups and using EXCEL when comparing only two groups. In this case, a one-tailed, two-sample, *t* test with unequal variances was used. Differences were considered significant at *p < 0.05.

## Results

### Effect of P/PL vs. HS culture on expression of myogenic markers

Traditional 2D monolayer culture was used to culture bmMSCs as this is the most widespread technique for MSC expansion [57]. As P/PL is the most widely used serum supplement for bmMSCs [38], bmMSCs were expanded in P/PL [14, 15]. We previously characterized primary MSCs expanded in P/PL and showed that these bmMSCs possess the basic phenotype CD73+/CD90+/CD105+ and tri-lineage differentiation potential [14, 58] as defined by the International Society for Cellular therapy [59] for MSCs. At passage 2 (day 0), bmMSCs were split and further cultured in P/PL or in HS which was successfully used to differentiate ADSCs, ucMSCs, and murine C2C12 cells following expansion in FBS-containing media [18, 32–34, 43].

**Fig. 1:**
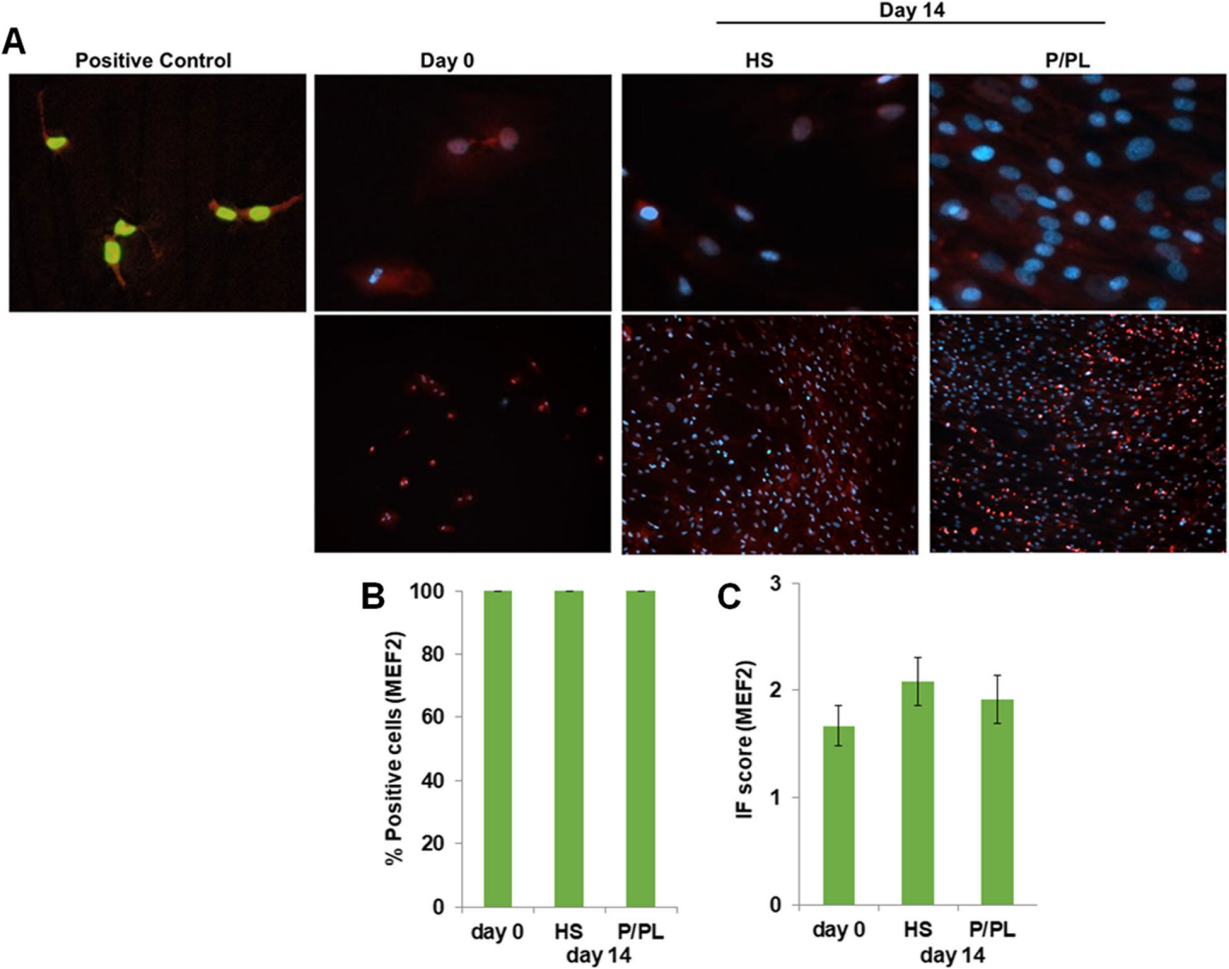
BmMSCs constitutively express MEF2 protein in all conditions tested. **a** Immunofluorescent staining of human myoblast SJCRH30 cells (Pos control; representative of *n* = 2, image dimension 1249 μm × 1102 μm) showing nuclear expression of MEF2. Day 0 (representative of *n* = 7 donors), day 14 post-HS (representative of *n* = 4 donors), and post-P/PL (representative of n = 7 donors) treatment demonstrate nuclear expression. Day 0 and day 14 image dimensions: 254 μm × 220 μ μm (upper panel) and 1249 μm × 1102 μm (lower panel). **b** Percent MEF2 positive cells. **c** Immunofluorescent score

**Fig. 2:**
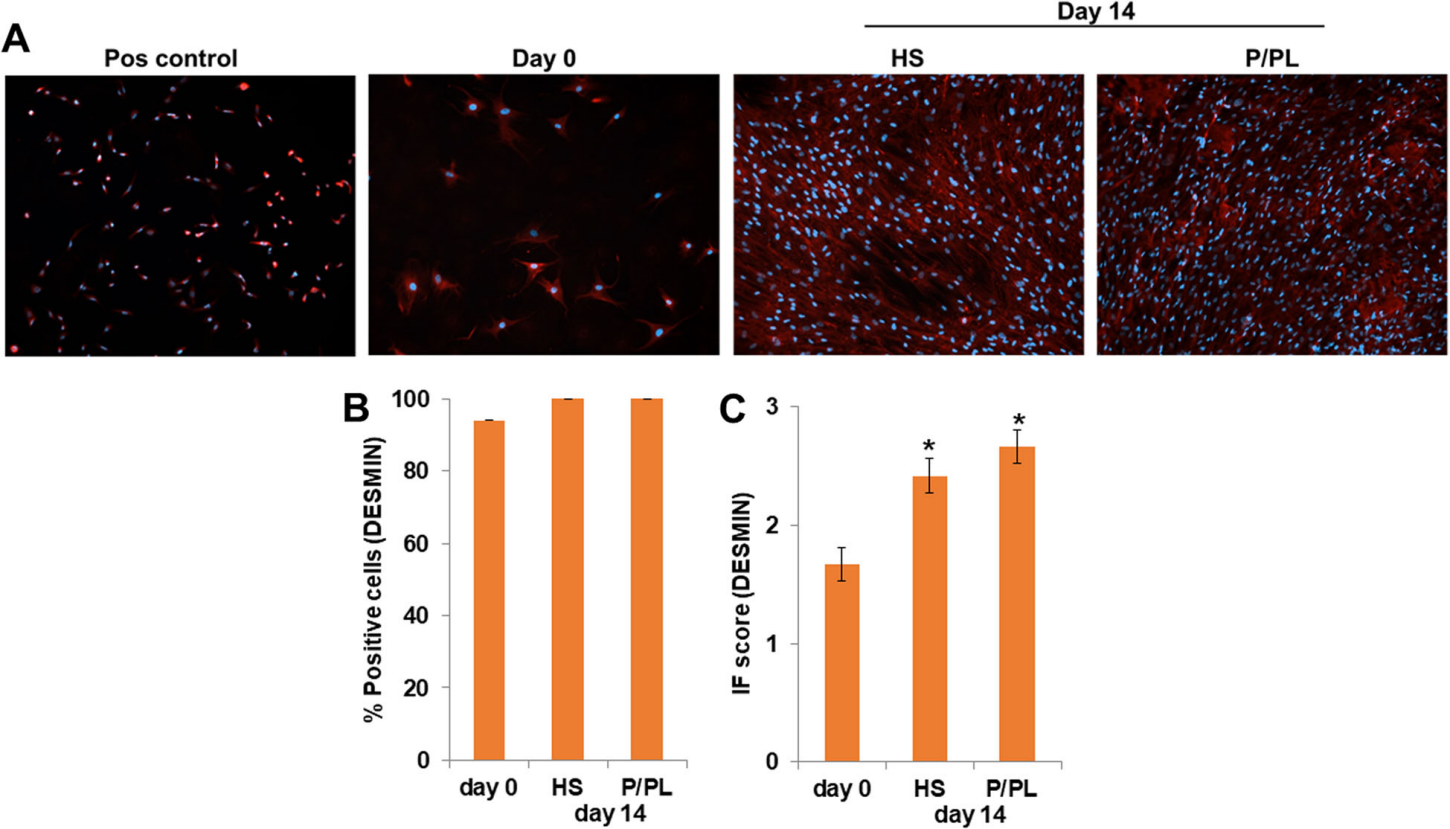
BmMSCs constitutively express Desmin protein, and HS and P/PL significantly increase the IF score of Desmin at day 14. **a** Immunofluorescent staining of human myoblast SJCRH30 cells (Pos control; representative of *n* = 2), day 0 (representative of *n* = 7 donors), day 14 post-HS (representative of *n* = 4 donors), and post-P/PL (representative of *n* = 8 donors) treatment. Image dimensions, 1249 μm × 1102 μm. **b** Percent Desmin positive cells. **c** Immunofluorescent score. *p < 0.05 vs. day 0 (ANOVA)

Myocyte enhancer factor 2 (MEF2) protein, a skeletal muscle transcription factor that participates in the coordinated regulation of genes during myogenesis [60–62], was constitutively expressed after expansion using P/PL (day 0) (Fig. 1a) as all cells were already positive for MEF2 at this stage (Fig. 1b). No significant differences in the percent positive cells (Fig. 1b) or IF score (Fig. 1c) between the tested conditions were detectable at day 14. Next, we investigated the expression levels of myogenic markers with ddPCR. In sharp contrast to human myoblasts (SJCRH30 cells) or human skeletal muscle positive controls, P/PL expansion (day 0) followed by treatment of bmMSCs with either P/PL or HS for 14 days led to very low mRNA (less than 2) copy numbers per microliter of the myogenic markers MyoD, MyoG, Myf5, and ACTA1 (Table 1), suggesting that these markers were not expressed under the chosen conditions. As others have shown that MyoD and MyoG increase early during MSC differentiation (e.g., up to 7 days) and not at later time points [18, 19, 32], we additionally investigated the expression of MyoD and MyoG at days 3 and 7 to ensure that we had not missed the expression of these markers. Similar to day 14, treatment of bmMSCs with P/PL or HS did not result in the expression of MyoD or MyoG myogenic markers. However, the later marker Desmin was expressed on the mRNA and protein level after expansion using P/PL (day 0) (Table 1 and Fig. 2a, respectively). At day 14, P/PL or HS did not further increase its gene expression compared to day 0 (Table 1). Desmin gene expression levels were much lower than the skeletal muscle positive control (Table 1). Immunofluorescent staining showed that human myoblast positive control cells also expressed Desmin and were considerably smaller in size than MSCs (Fig. 2a). These cells are approximately 10 μm in diameter [55] vs. MSCs which are generally 20–30 μm in diameter [56]. At day 0, between 72 and 100% of the cells were positive for Desmin (Fig. 2b). Levels reached 100% with further P/PL or HS cultures. Both P/PL and HS significantly increased the IF score of Desmin at day 14 vs. day 0 (Fig. 2c).

**Table 1:**
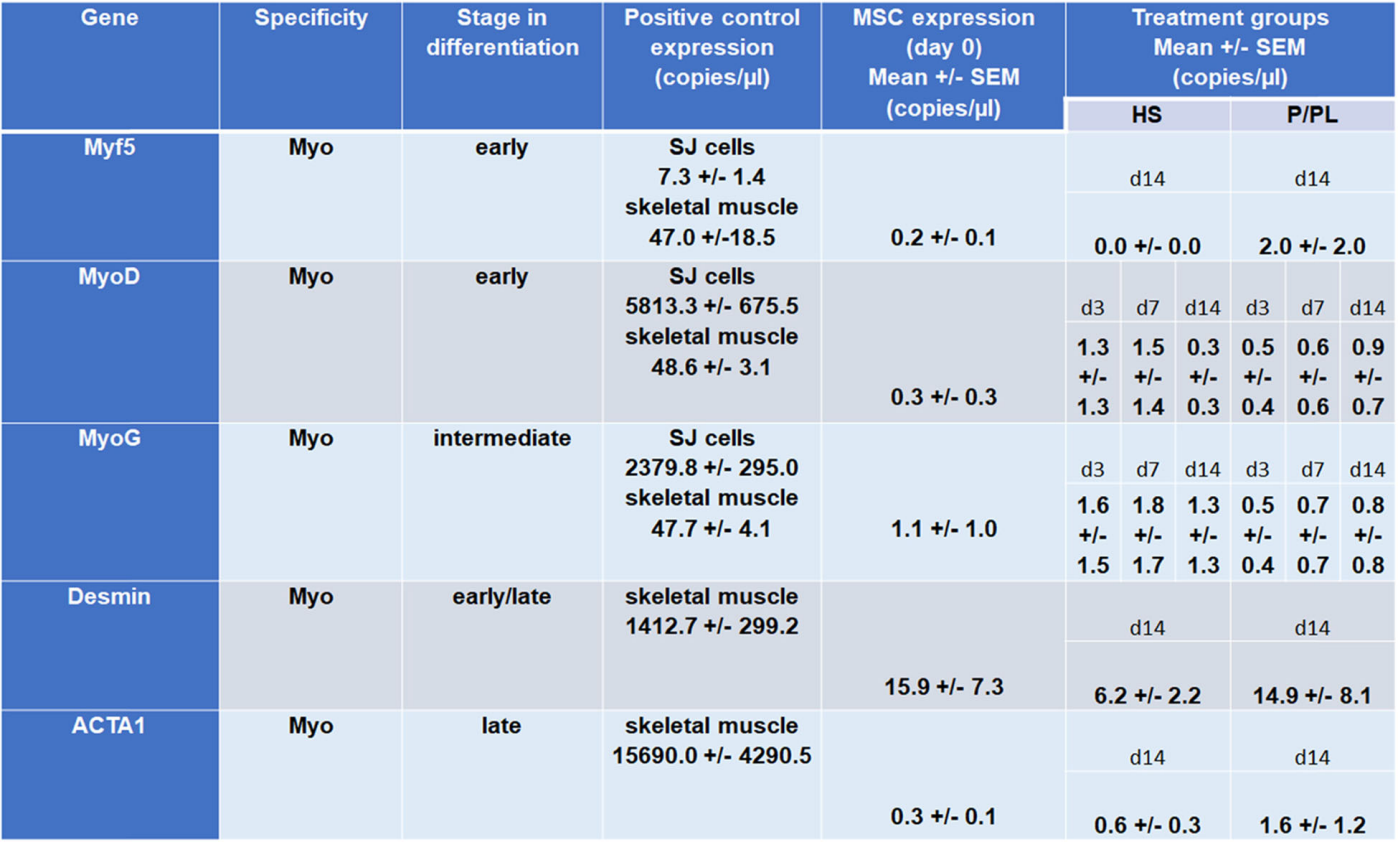
Lack of a skeletal muscle phenotype in adult human bone marrow stromal cells. The gene transcript levels are shown for the human myoblast SJCRH30 (SJ) cells and/or adult skeletal muscle (positive controls), bmMSCs at day 0 (after expansion in P/PL at passage 2) and following 14 days of treatment with either HS or P/PL. Data is expressed as mean (copies/μL) ± SEM of *n* = 4–9 donors/group

### Effect of P/PL vs. HS culture on expression of neurogenic markers

**Fig. 3:**
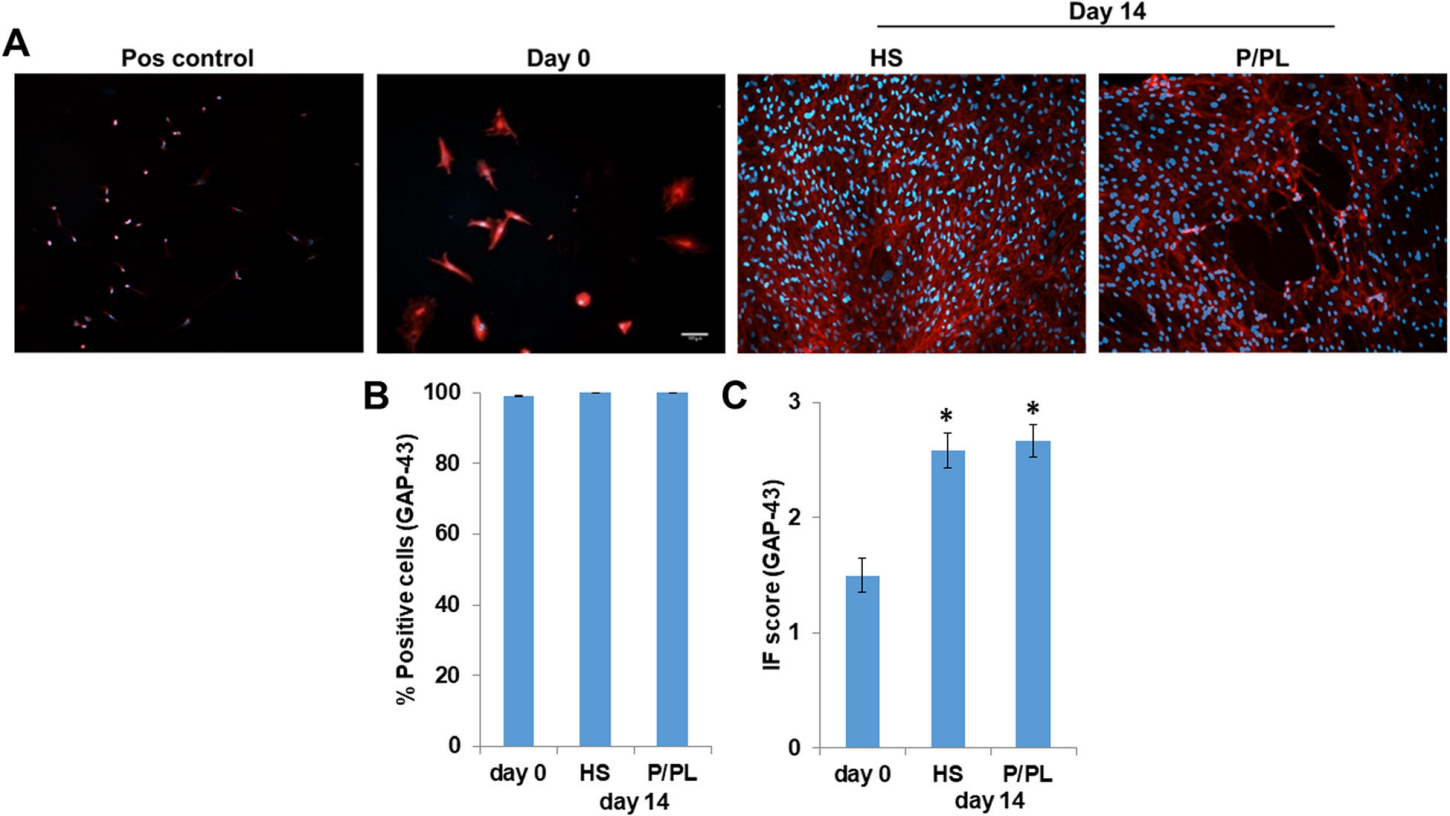
BmMSCs constitutively express GAP-43 protein, and HS and P/PL significantly similarly increased the GAP-43 IF score at day 14 compared to day 0. **a** Immunofluorescent staining of human myoblast SJCRH30 cells (Pos control; representative of *n* = 2), day 0 (representative of *n* = 8 donors), day 14 post-HS (representative of *n* = 4 donors), and post-P/PL (representative of *n* = 8 donors) treatment. Image dimensions, 1249 μm × 1102 μm. **b** Percent GAP-43 positive cells. **c** Immunofluorescent score. *p < 0.05 vs. day 0 (ANOVA)

Next, we measured the effect of HS vs. P/PL on neurogenic marker expression at day 0 and day 14. On the protein level, P/PL and HS resulted in equally high expression levels of the neurogenic growth associated protein 43 (GAP-43) at day 14 compared to day 0, and hence, all cells were positive for GAP-43 (Fig. 3b). P/PL and HS similarly significantly increased the IF score of GAP-43 at day 14 compared to day 0 (Fig. 3c). Interestingly, the expression was primarily observed in the cytoplasm as opposed to human myoblast SJCRH30 positive control cells, which showed more of a nuclear localization (Fig. 3a) similar to another study investigating GAP-43 expression in myoblasts [63].

**Fig. 4:**
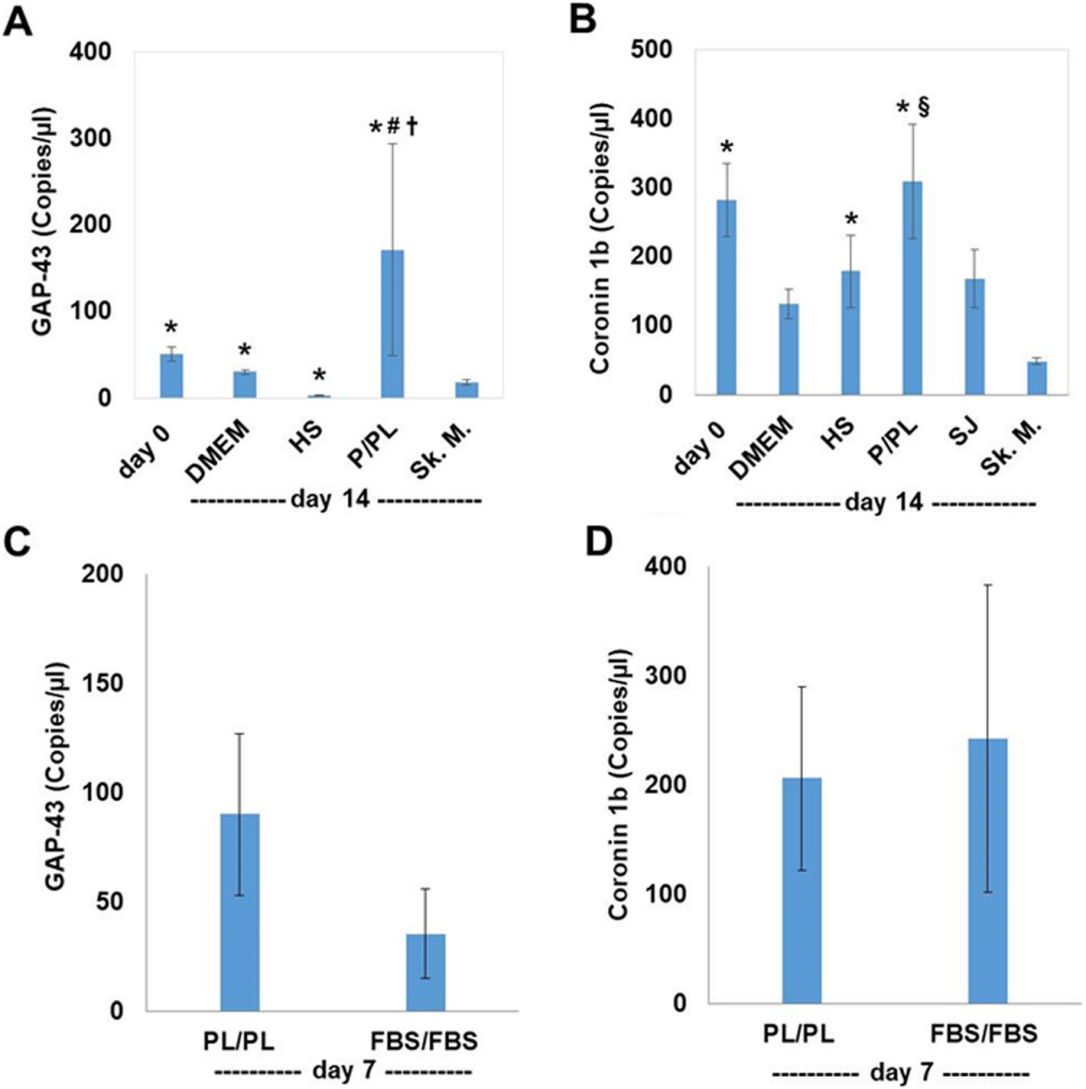
Neurogenic markers GAP-43 and Coronin 1b increase on the gene level when cultured in P/PL or FBS. **a** P/PL significantly increased the gene expression of GAP-43 at day 14. **b** Coronin 1b gene expression was highly expressed at day 0 levels and was maintained in P/PL or HS culture conditions at day 14. Data is expressed as mean (copies/μL) ± SEM of *n* = 4–9 donors/group. **c** P/PL and FBS similarly increased the gene expression of GAP-43 and Coronin 1b at day 7. Data is expressed as mean (copies/μL) ± SEM of *n* = 3–6 donors/group. The human myoblast SJCRH30 (SJ) cells and/or adult skeletal muscle (Sk.M.) was used as a control. *p < 0.05 vs. Sk. M. (*t* test); ^§^*p* < 0.05 vs. SJ cells (*t* test); ^#^*p* < 0.05 vs. day 0 (ANOVA); ^†^*p* < 0.05 vs. HS (ANOVA)

Compared to the human skeletal muscle positive control, day 0 bmMSCs, as well as day 14 DMEM and P/PL-treatment groups, expressed significantly higher levels of the GAP-43 gene (Fig. 4a). In contrast, the use of HS resulted in a significant decrease in the expression of GAP-43 compared to skeletal muscle. Analyzing the data over time, DMEM alone at day 14 resulted in similar levels of GAP-43 compared to day 0. P/PL, however, led to high levels of GAP-43 at day 14 with a 4.0 ± 1.7-fold increase in its mRNA expression compared to day 0. Moreover, P/PL resulted in a significant increase in the expression of GAP-43 compared to HS, and in fact, HS significantly decreased the expression of GAP-43 compared to day 0 resulting in only a 0.12 ± 0.02 fold change. P/PL expansion also led to a high baseline expression of the neurogenic gene Coronin 1b, which was maintained in P/PL or HS culture conditions (Fig. 4b). Furthermore, in comparison to human skeletal muscle, Coronin 1b expression was significantly increased in day 0 bmMSCs and day 14 HS- and P/PL-treatment groups. P/PL at day 14 also demonstrated significantly more expression of Coronin 1b than human myoblast SJCRH30 (SJ) cells. We subsequently investigated if the expression of these neurogenic markers was a serum-based phenomenon. For this experiment, bmMSCs were expanded in either FBS or P/PL and then further cultured in FBS or P/PL, respectively. Both conditions resulted in high levels of GAP-43 (Fig. 4c) and Coronin 1b expression (Fig. 4d) suggesting that it is a serum-based phenomenon.

### Effect of P/PL expansion on expression of neural cell adhesion molecule

**Fig. 5:**
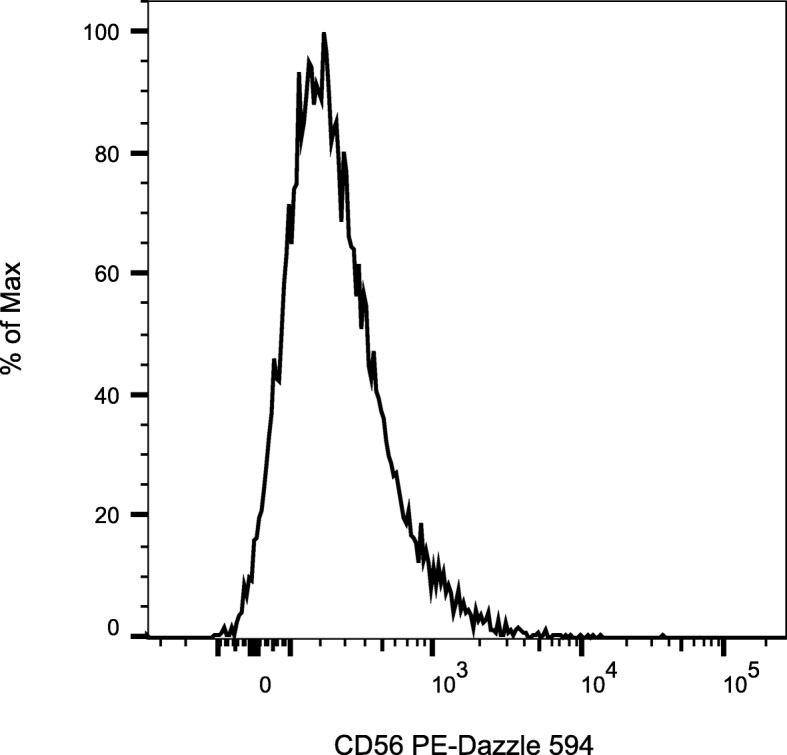
Passage 4 MSCs express CD56/NCAM following P/PL expansion. Bold histograms display staining with the CD56/NCAM antibody. Representative histogram of *n* = 3 MSC donors

As CD56 or neural cell adhesion molecule (NCAM) is considered a marker of neural lineage commitment [64, 65], we performed flow cytometry to determine if MSC cell populations were positive following P/PL expansion. Results demonstrated that the proportion of positive cells was 5.9 ± 2.1 (Fig. 5). This data suggests that bmMSC preparations in P/PL may contain a small portion of cells committed to the neural lineage.

## Discussion

Cell therapy is the fastest growing area of regenerative medicine with stem cell therapies currently listed as one of the largest areas of development which is expected to triple in size by the year 2025 [66]. The therapeutic potential and attractive properties of MSCs are evident from the increasing number of ongoing or upcoming clinical trials using human MSCs. As the cell therapy industry continues to develop, there is a significant need for critical analysis of the raw materials used in the production of MSCs and their effects on the differentiation potential of MSCs. Our work focused on the use of the human supplement P/PL on the expression of (neuro)myogenic markers and its ability to affect the differentiation potential of human bmMSCs towards a skeletal (neuro)muscular cell. Using the highly sensitive ddPCR method for detecting gene expression [44, 45], we show that expansion and culture of bmMSCs in P/PL did not result in the mRNA expression of myogenic genes (Myf5, MyoD, MyoG, and ACTA1). Moreover, we demonstrated that the use of the commonly used HS protocol for differentiating various cell types into myogenic cells [18, 32–34, 43] was unable to induce human bmMSCs into a myogenic phenotype following the expansion of bmMSCs in P/PL.

However, P/PL may have primed lineage determination as neurogenic markers were increased following P/PL expansion, as was Desmin. Hence, Desmin expression was already expressed after P/PL expansion and its protein score was significantly increased by further culture of bmMSCs in either P/PL or HS at day 14 compared to day 0, which is likely due to an increased number of cells. Others have shown that human bmMSCs express Desmin after FBS expansion [67, 68]. Nevertheless, Desmin is not specific to skeletal myogenic differentiation, as it is involved in early formation of cardiac and smooth muscle as well [69]. Our earlier studies emphasize this [14, 15]. We previously showed that expanding bmMSCs in human P/PL led to a baseline expression of several smooth muscle genes and proteins including ACTA2 (alpha smooth muscle actin), TAGLN (transgelin), and CNN1 (calponin). When bmMSCs were split and then further cultured in P/PL for 7 to 14 days, these markers were still present and sometimes even increased. While it appeared that expansion or further culture of bmMSCs in P/PL caused spontaneous differentiation of bmMSCs as demonstrated by the presence of these markers, they did not reach full functional maturity [15]. Moreover, the bmMSCs did not express the late smooth muscle myosin heavy chain (SM-MHC), which is the most mature smooth muscle cell phenotypic marker that allows smooth muscle cells to generate force along actin filaments [70, 71]. In contrast, addition of smooth muscle-inducing factors TGFβ1 and PDGF and ascorbic acid to P/PL-containing media caused bmMSCs to express significantly higher levels of these smooth muscle cell markers compared to day 0 and induced a major cell population having contractile function with 60–80% of the bmMSCs reaching functional maturity which exceeded the contractile function of bladder smooth muscle cells from elderly individuals. Hence, our previous studies showed that P/PL appeared to have primed the bmMSCs towards a smooth muscle cell phenotype, likely due to the high level of growth factors within P/PL (i.e., TGFβ1, PDGF) [39–42]. Our current study shows that P/PL under similar conditions does not prime adult bmMSCs towards the skeletal muscle lineage.

Interestingly, like Beier et al. [72], who expanded rat bmMSCs in FBS, we observed constitutive protein expression of MEF2 in the nucleus of all (100%) bmMSCs cultured in P/PL. MEF2 is a skeletal muscle transcription factor that participates in the coordinated regulation of genes during myogenesis [60–62]. However, as noted, we did not observe any expression of Myf5, MyoD, MyoG, and ACTA1. It is therefore likely that the bmMSCs in the present study did not undergo myogenesis indicated by the lack of expression of these other myogenic markers, as induction of skeletal myogenic differentiation depends on the activities of two groups of transcription factors. The first group, also known as myogenic regulatory factors (MRFs), includes Myf5, MyoD, MyoG, and MRF4. Expression of any of these MRFs converts non-myogenic mesenchymal cells into myoblasts [73]. While MRF4 was not investigated in the present study, importantly we did not observe any expression of MRFs MyoD or MyoG on the gene level. MEF2 belongs to the second family of transcription factors needed for myogenesis. It has been proposed that MEF2 and MRFs cooperate in a positive feedback loop to regulate myogenesis [62, 73]. Although we show that MEF2 was present in bmMSCs, the MRFs MyoG, MyoD, and MYF5 were not expressed. This suggests that although MEF2 is present in bmMSCs cultured in P/PL, due to the lack of MRFs, the coordinated effects of MRFs and MEF2 needed to stimulate the cells towards the skeletal muscle differentiation pathway cannot occur.

We do not dispute the fact that many murine and rat animal models have demonstrated that bmMSCs migrate into degenerating or damaged skeletal muscle and participate in regeneration and repair of skeletal muscle [16–30]. However, the mechanism by which human bmMSCs regenerate skeletal muscle remains unclear. Some studies suggest that bmMSCs may contribute to regeneration through their ability to secrete growth factors and cytokines which can modulate the inflammatory local response [74, 75]. Hence, one study suggested that skeletal muscle regeneration is due to bmMSCs acting as a reservoir of cytokines that aid in the migration and proliferation of inflammatory cells which could result in the sequential regeneration of myofibers [26]. This was confirmed by a recent study showing that bmMSCs function in regeneration of skeletal muscle by creating a macrophage M1/M2 balance which in turn promotes myoblast differentiation [29] or that bmMSCs stimulate growth factors such as VEGF which could aid in angiogenesis and satellite cell pool maintenance [29, 74]. Interestingly, a few studies have shown that the differentiation kinetics of bmMSCs differs from committed adult myogenic precursor cells [25, 76]. While injected satellite cells were able to fuse into damaged muscle fibers within 5 days, bmMSCs were only detected 2 weeks after muscle damage, suggesting a different time scale by which bmMSCs contribute to muscle regeneration. This was confirmed in another study that showed that enriched myogenic murine bmMSC clones remained dormant in culture for 10–15 days with a spherical shape and then began to rapidly multiply [76]. Therefore, bmMSCs appear to contribute to the regenerative process, but much more slowly than satellite cells. Moreover, the frequency of fusion of bmMSCs is low and there is considerable donor variability in the fusion of bmMSCs [25, 77, 78].

It is noteworthy to point out that others have shown that some human bmMSC cultures expanded in 10% FBS expressed MyoD, MyoG, and MHC as determined by PCR and/or immunofluorescence [79, 80]. Moreover, human placenta- [31], umbilical cord- [32], and adipose-derived MSC studies [33, 34] have shown that MSCs from these tissues are capable of undergoing skeletal muscle lineage commitment using FBS expansion followed by treatment with HS. Even after up to 28 days of culture in P/PL, we did not observe the expression of myogenic markers in human bmMSCs (data not shown). As we did not observe the expression of myogenic markers, with the exception of Desmin and MEF2, differences could be attributed to the growth factor supplement of FBS in their study vs. P/PL used in the present study or insufficient levels of growth factors such as bFGF, PDGF, forskolin, neuroregulin, IGF, and VEGF needed for driving the bmMSCs towards a myoblast [18, 19]. However, it may be more likely that the process of bmMSC skeletal muscle lineage commitment is more complex and may require Notch signaling at a defined time point as well as MSC secretory factors as Dezawa et al. have illustrated [18]. They showed that treatment of human bmMSCs with a combination of bFGF, PDGF, forskolin, and neuregulin for 3 days followed by subsequent gene transfection with the notch intracellular domain (NICD) induced MyoD expression in bmMSCs and caused a small percentage of cells to spontaneously contract. Moreover, the addition of either HS or MSC supernatant to these cells for 5 to 14 days caused the differentiated cells to fuse into myofibers which were capable of contributing to skeletal muscle repair in in vivo rat and mouse models. Importantly, when they reversed the order of cytokine treatment and NICD transfection, the bmMSCs could not under myogenesis as the bmMSCS were incapable of expressing the MRFs Myf5, MyoD, MyoG, and MRF4, similar to what we showed. Other studies support the importance of Notch signaling during the differentiation of MSCs into cardiomyocytes [81–83]. IGF signaling has also been shown to regulate the myogenesis of placenta MSCs as silencing of IGF binding proteins blocked lineage commitment in placenta-derived MSCs [31].

A limitation of our study was that myogenesis was largely investigated by the expression of myogenic markers on the transcript level. Using highly sensitive ddPCR [37, 38], we demonstrated that with the exception of Desmin, less than 2 copies/μL were detected for all of the myogenic markers measured, in sharp contrast to the human myoblast cell line or human skeletal muscle which showed a high number of positive droplet fractions and hence a high expression of myogenic markers (Table 1). As PCR shows only average transcript levels, a small or rare population of bmMSCs beginning to differentiate may be masked in bulk measurements. However, ddPCR is an absolute endpoint measurement, as the amount of synthesized transcript copies per microliter per gene is detected. Thus, while it is true that ddPCR detects the average level of transcript, the transcripts synthesized by a small population of differentiating cells should still be detectable due to the resolution of the technique [44–46]. This means the results are not masked in terms of being non-detectable, but masked in terms of resulting in a lower average of the entire population. As long as there is some expression, ddPCR should be capable of detecting the expressed transcript copies.

Our data does demonstrate that our bmMSC preparations may contain a portion of cells committed to the neural lineage. P/PL caused a significant increase in the early neural GAP-43 mRNA expression compared to HS and skeletal muscle (day 14). Moreover, after P/PL expansion, 99% of bmMSCs expressed GAP-43 and levels reached 100% at day 14 with further P/PL or HS culture. Furthermore, the IF score was significantly increased by both P/PL and HS as was observed after 14 days of culture and resulted in the formation of networks with neighboring cells. In addition to GAP-43’s role in the formation of neuromuscular junctions in skeletal muscle [63, 84, 85], its well-known role is in neurite (axonal) outgrowth during neurogenesis where it is also localized in the cytoplasm of those cells [86–89]. Our study showed that bmMSCs conditioned with P/PL also showed cytoplasmic GAP-43 expression, as opposed to the human myoblast SJCRH30 cell line which showed nuclear staining. This cytoplasmic expression is in line with other MSC studies that used bFGF or epidermal growth factor (EGF) to differentiate bmMSCs into functional neuronal cells expressing GAP-43 [90–93]. PL has been shown to contain high amounts of both bFGF and EGF, in addition to a number of other growth factors [39–42]. In addition to GAP-43 expression, we additionally demonstrated that bmMSCs expanded in P/PL expressed high levels of Coronin 1b, which belongs to the neuronal regeneration-associated genes involved in neurite outgrowth [86, 94]. Together, this suggests that P/PL may have a neurogenic-inducing effect on human bmMSCs. However, given that all cells were positive for GAP-43 at day 0, bmMSCs expanded in P/PL may partially be differentiating towards an early neurogenic-like cell type already at the expansion stage, in addition to differentiating bmMSC towards a smooth muscle cell-like phenotype as we previously have shown [14, 15]. As FBS expansion and further culturing resulted in equally high levels of GAP-43 and Coronin 1b, this data suggests that P/PL and FBS may contain a portion of cells committed to the neural lineage. Collectively, this shows that P/PL partially induces neurogenesis and smooth muscle myogenesis, but does not prime adult bmMSCs towards the skeletal muscle lineage.

However, the percentage of human bmMSCs committed to neurogenesis may be limited as only a small proportion (5.9 ± 2.1) of passage 4 bmMSCs expanded in P/PL were positive for the neural lineage commitment CD56/NCAM phenotypic marker [64, 65]. There are conflicting reports on the expression of CD56/NCAM on human bmMSCs with two studies showing that bmMSCs cultured in 10–20% FBS do not express or express very little CD56 [95, 96], while another study showed that the fraction of CD56^bright^ bmMSCs was less than 15% [97]. A study investigating bmMSCs from five healthy females (age 21–31 years old) expanded in human PL and assessed at passages 2 or 3 showed that the proportion of positive cells varied considerably with the percentage of positive cells extending from 23.6 to 88.5% [98]. In our study, bmMSCs were isolated mostly from an older population of donors undergoing hip surgery and expanded in P/PL as opposed to their study that used only human PL, suggesting that age or the addition of human (P) plasma to the media may alter the CD56 expression. As we only focused on the expression of GAP-43, Coronin 1b, and CD56/NCAM in the present study, follow-up experiments for assessing more specific markers of neurogenesis would be helpful, as well as electrophysiological responses to determine the extent of functionality of this small population of MSCs that may be committed to neurogenesis in the presence of P/PL.

## Conclusions

Collectively, despite bmMSCs expressing the MRF MEF2 and Desmin after expansion in P/PL, our study demonstrated that bmMSCs cultured in P/PL did not differentiate into skeletal precursors, as bmMSCs cultured in xenogeneic-free media such as P/PL did not induce the expression of other MRFs required for myogenesis and HS had no effect. However, P/PL to some extent appeared to have a neurogenic effect. Further investigations on the use of P/PL are warranted in more realistic human ex vivo models of myogenesis and neurogenesis.

## Data Availability

The datasets used and/or analyzed during the current study are available from the corresponding author upon reasonable request.

## Abbreviations

ADSC: Adipose-derived mesenchymal stromal cells
bFGF: Basic fibroblast growth factor
bmMSCs: Bone marrow-derived mesenchymal stromal cells
EGF: Epidermal growth factor
FBS: Fetal bovine serum
GAP-43: Growth associated protein 43
HS: Horse serum
IGF-1: Insulin-like growth factors
MEF2: Myocyte enhancer factor 2
MRFs: Myogenic regulatory factors
MSCs: Mesenchymal stromal cells
NCAM/CD56: Neural cell adhesion molecule
P: Plasma
PDGF: Platelet-derived growth factor
PL: Platelet lysate
S: Serum
ucMSCs: Umbilical cord-derived mesenchymal stromal cells
VEGF: Vascular endothelial growth factor
